# Supplementary material for: Gas Biosensor Arrays Based on Single-Stranded DNA-Functionalized Single-Walled Carbon Nanotubes for the Detection of Volatile Organic Compound Biomarkers Released by Huanglongbing Disease-Infected Citrus Trees
Source: Sensors (Basel). 2019 Nov 4;19(21):4795. doi: 10.3390/s19214795 (PMC6865013; doi:10.3390/s19214795)
Supplement: Supplementary file 1 [file sensors-19-04795-s001.pdf]

Supporting Information

Single-stranded DNA-Functionalized Single-Walled Carbon Nano-tubes Gas Biosensor Arrays for the Detection of Volatile Organic Compounds Biomarker released by the Huanglongbing Disease infected Citrus tree

Hui Wang [1,2,3,5,†], Pankaj Ramnani [3,†], Tung Pham [3], Claudia Chaves Villarreal [4], Xuejun Yu [3], Gang Liu [1,2,*] and Ashok Mulchandani [3,4,*]

[1] Key Laboratory of Modern Precision Agriculture System Integration Research, Ministry of Education China Agri-cultural University, Beijing 100083, China;

[2] Key Laboratory of Agricultural Information Acquisition Technology, Ministry of Agriculture China Agri-cultural University, Beijing 100083, China; wanghuilunwen@cau.edu.cn (H. W.)

[3] Department of Chemical and Environmental Engineering, University of California-Riverside, Riverside, CA 92521, USA; pramn002@ucr.edu (P.R.) tpham052@ucr.edu (T.P.); xyu010@ucr.edu (X.Y.)

[4] Materials Science and Engineering Program, University of California-Riverside, Riverside, CA 92521, USA; cchav021@ucr.edu

[5] Research Institute of Wood Industry, Chinese Academy of Forestry, Beijing 100091, China.

* Correspondence: pac@cau.edu.cn (G.L.); adani@engr.ucr.edu (A.M.); Tel.: +86-10-6273-6741 (G.L.); +1-951-8276419(A.M.)

† Equal Contribution.

Received date: 9 October 2019; Accepted date: 1 November 2019; Published: date

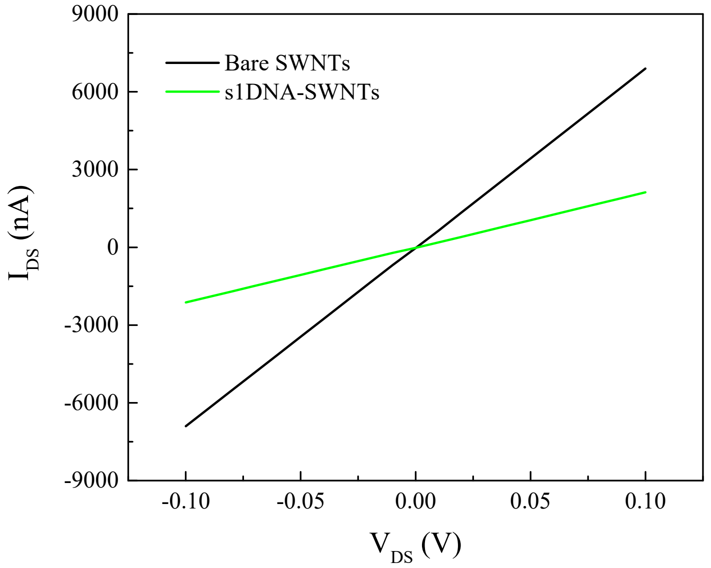

Figure 1. I_{DS}-V_{DS} characteristics of SWNTs before and after functionalization with s1DNA at V_G=0 V.

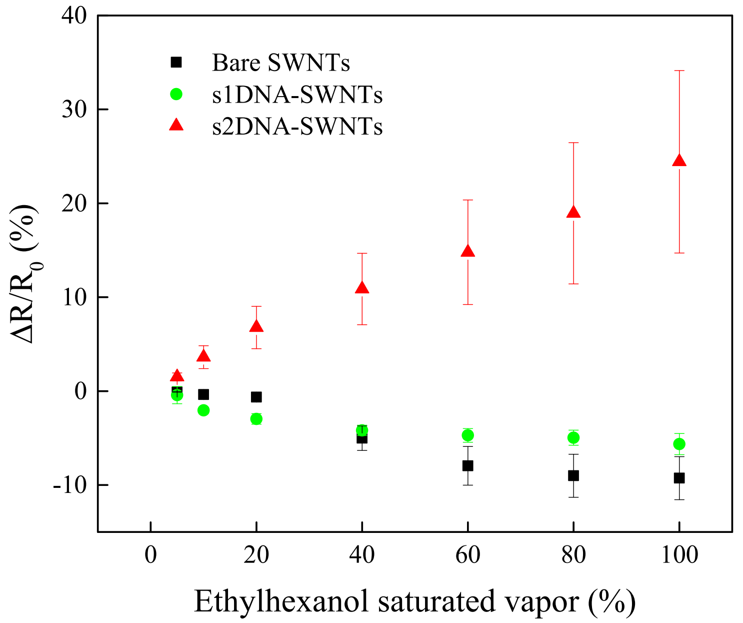

Figure 2. Calibration curves of bare and ssDNA coated SWNTs sensor towards different concentrations of ethylhexanol vapors performed at V_{DS}=0.1 V.

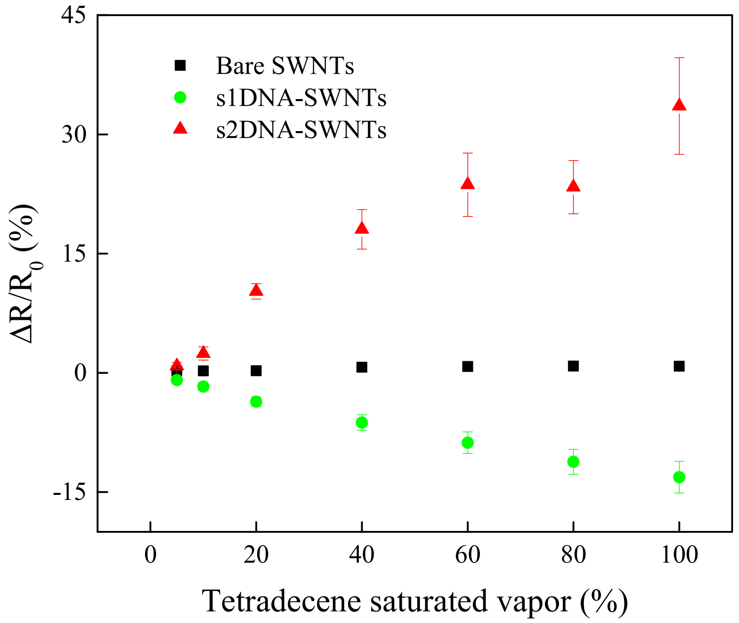

Figure S3. Calibration curves of bare and ssDNA coated SWNTs sensor towards different concentrations of tetradecene vapors performed at V_{DS}=0.1 V

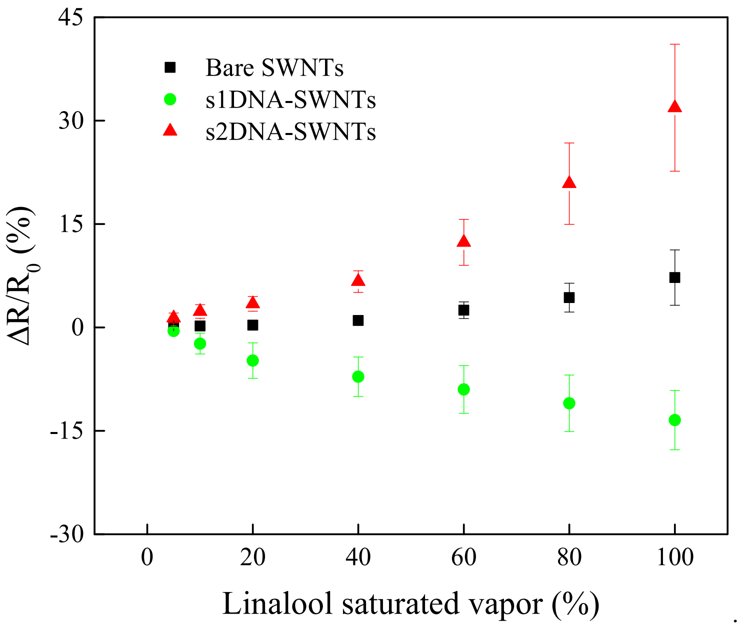

Figure 4. Calibration curves of bare and ssDNA coated SWNTs sensor towards different concentrations of linalool vapors performed at V_{DS}=0.1 V.

In this experiment, we use the gas device to generate the different VOCs. Due to the different vapor pressures of the different materials, the certain concentrations are totally different. It is much convenient for us using the percent concentration.

$$PV=nRT$$

P = Pressure (atmospheres), V = Volume (liters), n = Number of moles of gas, R = Molar gas constant, T = Temperature (Kelvin)

Table 1. The concentrations of four VOCs at 25 °C.

	Analyte	Physical	CAS	Vapor Pressure	Concentration	LOD
1	Ethylhexanol	liquid	104-76-7	0.36 mmHg	432.72	7.005 ppm
2	Phenylacetaldehyde	liquid	122-78-1	0.368 mmHg	442.336	6.21 ppm
3	Linalool	liquid	78-70-6	0.0905 mmHg	108.781	1.24 ppm
4	(1-)tetradecene	liquid	1120-36-1	0.01 mmHg	12.02	0.104 ppm

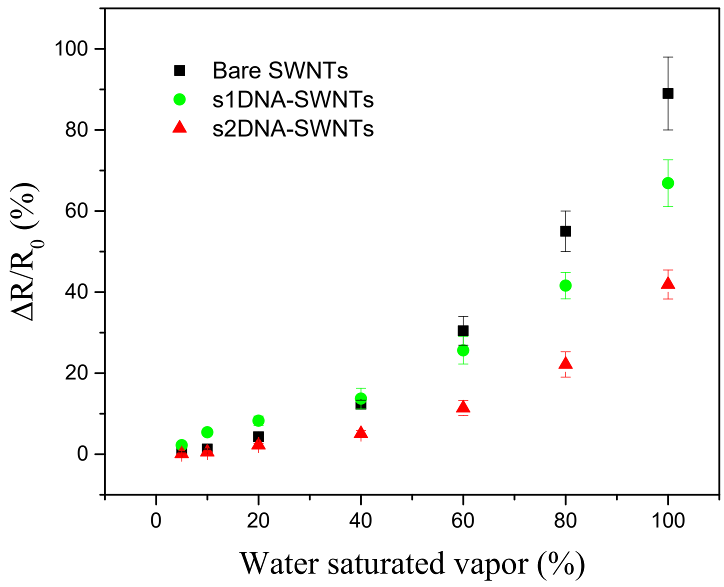

Figure S5. Calibration curves of bare and ssDNA coated SWNTs sensor towards different concentrations of water vapors performed at V_{DS}=0.1 V

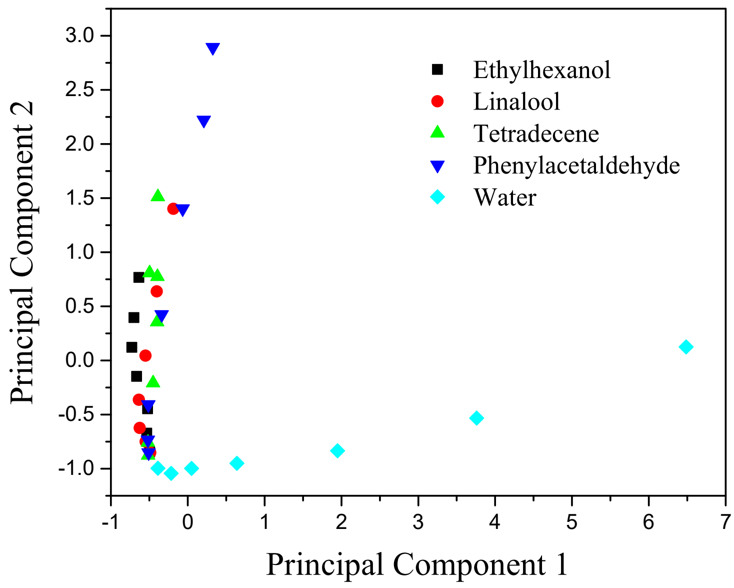

Figure 6. PCA plot (PC1 vs. PC2) of scores using three sensors (bare SWNTs, s1DNA-SWNT and s2DNA-SWNT) for four VOCs and water teste.

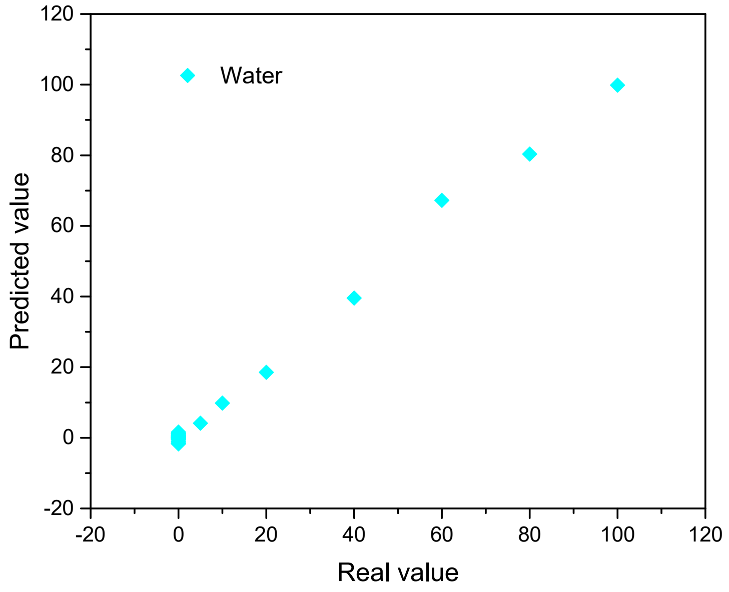

Figure 7. The real value versus the predicted value towards the different concentrations of water vapor calculated by the NNF.
